# Toxicity of Difenoconazole and Atrazine and Their Photodegradation Products on Aquatic Biota: Environmental Implications in Countries Lacking Good Agricultural Practices

**DOI:** 10.3390/toxics11030213

**Published:** 2023-02-24

**Authors:** Julia Mendieta Herrera, Carlos Iñiguez Armijos, Daniel Rosado Alcarria, Silvio Aguilar Ramírez

**Affiliations:** 1 Departamento de Química y Ciencias Exactas, Universidad Técnica Particular de Loja, San Cayetano Alto s/n, Loja 1101608, Ecuador; 2 Departamento de Ciencias Biológicas y Agropecuarias, Universidad Técnica Particular de Loja, San Cayetano Alto s/n, Loja 1101608, Ecuador; 3 Department of Hydrology and Water Resources Management, Institute for Natural Resource Conservation, Kiel University, 24118 Kiel, Germany

**Keywords:** *Lemna minor*, *Daphnia magna*, pesticides, aquatic toxicity

## Abstract

Agriculture is fundamental for human development, but it may also have a range of unwanted effects on ecosystems when pesticides inadvertently enter the environment. We determined the toxicity of difenoconazole and atrazine, as well as their photodegradation products, on the bioindicators *Lemna minor* and *Daphnia magna*. For *L. minor*, we assessed the number of leaves, biomass, and chlorophyll content exposed to different concentrations of difenoconazole (0–8 mg/L) and atrazine (0–3.84 mg/L). For *D. magna*, we assessed the mortality to difenoconazole (0–1.6 mg/L) and atrazine (0–80 mg/L). We found that the higher the concentrations of the pesticides, the higher the toxicity for both bioindicators. In *L. minor*, the highest toxicity for atrazine was 0.96 mg/L, whereas for difenoconazole, it was 8 mg/L. For *D. magna*, the 48 h LC_50_ for difenoconazole was 0.97 mg/L, while for atrazine, it was 86.19 mg/L. For *L. minor*, the toxicity of difenoconazole and atrazine was not different compared to that of their photodegradation products. In contrast, for *D. magna*, difenoconazole, but not atrazine, was more toxic compared to its respective photodegradation products. Pesticides are a serious threat to aquatic biota, and their photodegradation products remain toxic in the environment. Additionally, the use of bioindicators can help monitor these pollutants in aquatic ecosystems in countries where the application of pesticides is imperative for agricultural production.

## 1. Introduction

The contamination of aquatic ecosystems worldwide is a drastic issue, which worsens every day by the inadvertent entry of pesticides into the environment [1]. Only very low percentages of applied pesticides reach the target plant, and most pesticides simply end up in aquatic ecosystems through percolation, evaporation, leaching, runoff, and erosion [2]. Thus, it is almost impossible to trace the flow of pesticides in the environment.

Difenoconazole and atrazine stand out as being among the most used pesticides in agriculture that have been detected as contaminants in the environment [3,4]. Difenoconazole (triazole family) is a fungicide that interferes with the biosynthesis of ergosterol in fungi, acting mainly on the demethylation of C^14^, which causes morphological and functional alterations of the cell wall [4]. In addition, it belongs to the group of endocrine disruptors, which are known to cause damage to human health and cause acute and chronic toxicity in aquatic environments (H400 and H410, respectively, according to the classification of the Globally Harmonized System of Classification and Labelling of Chemical Products (GHS)) [5,6]. Atrazine (triazine family) is a herbicide that inhibits photosynthetic electron transport in leaves [4]. It is leached through the soil by rain or irrigation water until it reaches bodies of water, where it is frequently detected due to its low solubility in water [5]. In addition, atrazine is listed as hazardous [6] (acute and chronic toxicity, H400 and 410, respectively) for aquatic environments.

Pesticides present in the environment are degraded by physicochemical (e.g., photodegradation by solar irradiation) or biological processes (e.g., degradation by microbial activity), giving rise to transformation products. In many cases, these transformation products have unknown effects on the environment [7], are known to cause loss of species [7], or have been classified as carcinogenic, neurotoxic, and teratogenic [8]. Some authors, such as Man et al. [9], found that photodegradation products of difenoconazole are toxic for fish but not for crustaceans. Klementová et al. [3] reported no toxicity of the combined photodegraded products of atrazine on aquatic plants, while crustaceans only suffered toxic effects after long-term exposure to atrazine. Evgenidou and Fytianos [10] investigated the transformation products of atrazine via different degradation forms. However, it is still necessary to understand how these compounds affect water bodies, their degradation pathways, the means of detection, the effects of chronic exposure, and the responses of aquatic biota.

Toxicity bioassays with biological indicators are already standardized techniques (Standard Methods 8211), which tests give reliable and reproducible/repeatable results [11]. The use of biological indicators in toxicity tests is on the rise due to the ease of installation, maintenance, and adaptation to laboratory conditions, which lowers the costs of studies [12]. *Lemna minor* is a floating macrophytic aquatic plant that inhabits freshwater bodies; it is commonly used in aquatic ecotoxicity tests and is recommended for toxicity evaluations in processes where pesticides are used [13,14]. Within the animal kingdom, *Daphnia magna* is the most commonly used cladoceran crustacean in ecotoxicological tests because it is easy to establish in the laboratory and has a short life cycle [15]. The USEPA [11,16] recommend the use of these species for the evaluation of agrochemical toxicity in aquatic environments.

Agricultural production in Ecuador contributes 8% to the country’s total annual production [17]. According to the FAOSTAT data, Ecuador registered pesticide use of approximately 14.03 kg/ha in 2019 [18]. Difenoconazole is applied to control Black Sigatoka caused by the fungus *Mycospharella fijensis* in banana crops, and it is an agrochemical considered to be moderately dangerous (II) according to the toxicological category of the World Health Organization [8,17,19]. Atrazine, on the other hand, is applied as a weed controller in corn and sugar cane crops and it is an agrochemical considered to be slightly dangerous (III) [10,20]. In Ecuador, the areas planted with bananas occupy 165,080 ha, and the areas planted with sugarcane occupy more than 157,900 ha [17].

Therefore, the present study investigated the toxic effects of difenoconazole and atrazine and the potential toxicity of their photodegradation products on aquatic biota by using *L. minor* and *D. magna* as bioindicators. The photodegradation products were obtained by exposing both pesticides to UV irradiation in the laboratory. As the response variables, we used the number of leaves, biomass, and chlorophyll content of *L. minor* and percentage of mortality of *D. magna*. We expected that the toxicity of the photodegradation products is lower than that of the pesticides.

## 2. Materials and Methods

### 2.1. Difenoconazole and Atrazine Photodegradation

We used commercial-grade difenoconazole (Score^®^ 250 EC, Syngenta S.A., Cartagena, Colombia) and commercial-grade atrazine (ATRAPAC^®^ 900, Agripac, Guayaquil, Ecuador). The photodegradation products were obtained by exposing both pesticides to irradiation with a 6 W UV-C light lamp (200 to 280 nm) in a cylindrical reactor (Microfilter Ultraviolet Sterilization Filter model OPP-625 1.0 GPM. Sejong, Republic of Korea) with an adjustable flow rate peristaltic pump (Cole Parmer model 7523-80 Masterflex L/S, Waltham, MA, USA).

The photodegradation conditions for both pesticides were as follows: for difenoconazole, a 8 mg/L solution was prepared and then irradiated for 2 and 4 min, whereas for atrazine, a 3.84 mg/L solution was prepared and then irradiated for 15 min until photodegradation products were obtained (Table 1). Pesticide concentrations and exposure times (irradiation with UV-C light lamp) were established after performing and monitoring previous tests until photodegradation products were detected through high performance liquid chromatography (HPLC).

All tests during the assays were carried out with the previously mentioned commercial pesticides; for their analytical determination, we used the standard solutions for difenoconazole (Difenoconazol PESTANAL^®^, Sigma Aldrich, St. Louis, MO, USA) and atrazine (Atrazine PESTANAL^®^, Sigma Aldrich, St. Louis, MO, USA).

The analytical determination of difenoconazole, atrazine, and their photodegradation products was performed using a HPCL coupled to a mass spectrometer (Bruker amaZon Ion Trap Mass, Bremen, Germany) and a UV detector (Thermo Scientific, Dionex UltiMate 3000 modular system, Waltham, MA, USA).

### 2.2. Toxicity Tests with Lemna Minor

The toxicity tests for *L. minor* were carried out according to the guidelines of the Standard Methods 8211 [11]. Prior to the tests, individuals of *L. minor* (adapted and maintained under laboratory conditions [11]) were cultured for two weeks in a nutrient solution for duckweed (see Table S1 for details about solution) and simultaneously subjected to an adaptation period with the light (24 h; 2150–4300 lux) and temperature (24 ± 2 °C) conditions used during the toxicity tests. We prepared six concentrations of difenoconazole (0, 0.5, 1, 2, 4, and 8 mg/L) from a stock solution of difenoconazole (1000 mg/L), and six concentrations of atrazine (0, 0.12, 0.24, 0.48, 0.96, 1.92, and 3.84 mg/L) from a stock solution of atrazine (192 mg/L). To test the effect of the photodegradation products from both pesticides, individuals of *L. minor* were exposed to a solution of 8 mg/L of difenoconazole and to a solution of 3.84 mg/L irradiated with UV-C light. The selection of these concentrations was based on previous range-finder tests, where both bioindicators were exposed to different concentrations of each pesticide until we detected damage or alteration in the exposed organisms to select a reference initial concentration. Then, from the initial concentration, we successively increased the concentrations by two-fold.

Three replicates were created for each concentration of the pesticides and their photodegradation products. In each replicate, we placed 10 healthy individuals of *L. minor* in a 350 mL glass bowl containing 100 mL of the nutrient solution and an aliquot of each concentration. *L. minor* individuals were similar in size and composed of 2 to 3 leaves; they were exposed to the pesticides and photodegradation products for 7 days. During the exposure period, the medium was not refreshed. Once the exposure time elapsed, the individuals of *L. minor* were carefully removed and washed with DI water to determine the average total number of leaves, the average total biomass, and the average total chlorophyll content per replicate, as explained below.

The total number of leaves was counted at the beginning and at the end of the exposure time (7 days). A new leaf was determined as every shoot that was observed in the individuals. Any sign of chlorosis or deterioration in the leaves was also recorded. Then, the number of leaves and the total biomass were determined. The individuals were placed in 1.5 mL Eppendorf tubes that had been previously waxed and covered with parafilm, making 3 to 4 perforations. Then, the individuals were lyophilized for a period of 3 hours at −50 °C and 0.250 mBar (Labconco modelo 7754047. Kansas City, MO, USA) and weighed to the nearest 0.0001 mg. Once weighed, the individuals of *L. minor* were placed in centrifugation tubes (10 mL) with 2 mL of 80% acetone. Subsequently, they were placed in a water bath at 25 °C in the dark for 24 h, with continuous agitation for the extraction of chlorophyll Then, the chlorophyll concentration was quantified in a spectrophotometer (HACH DR 2800, Düsseldorf, Germany) by measuring the absorbance at 665 nm (Chl-*a*) and at 649 nm (Chl-*b*), respectively. We chose these absorbance values based on the literature [21,22] and on previous tests carried out in the laboratory after the maximum absorbance was determined.

### 2.3. Toxicity Tests with Daphnia Magna

The toxicity tests for *D. magna* were carried out based on the protocols of the Standard Methods 8711. Prior to the tests, individuals of *D. magna* were cultured for four weeks in a medium consisting of reconstituted hard water according to the Standard Methods 8010:1 (Table S2). During this time, the individuals of *D. magna* (adapted and maintained in the laboratory facilities [11]) were adapted to the light (16 h; 528–1076 lux) and temperature (20 ± 2 °C) conditions used in the toxicity tests. We prepared six concentrations of difenoconazole (0, 0.1, 0.2, 0.4, 0.8, and 1.6 mg/L) from a stock solution of difenoconazole (10 mg/L). For atrazine, we also prepared six concentrations (0, 10, 20, 40, 60, and 80 mg/L) from a stock solution of atrazine (500 mg/L). To test the effect of the photodegradation products from both pesticides, the individuals of *D. magna* were exposed to a solution of 1.6 mg/L of difenoconazole and to a solution of 80 mg/L of atrazine irradiated with UV-C light. The selection of these concentration was similarly made as explained above.

For each concentration of the pesticides and their photodegradation products, three replicates were created by placing 10 neonates (i.e., 24 h after hatching) of *D. magna* in a 250 mL beaker containing 50 mL of reconstituted hard water plus the aliquot of each concentration per replicate. The *D. magna* neonates were exposed to the pesticides and photodegradation products since hatching for 48 h. Once the exposure time was completed, the living and immobile individuals were quantified through observation. Then, immobility was used as a proxy of mortality, and its percentage was calculated.

### 2.4. Data Analysis

Data analysis was performed in the R programming environment version 4.1.3 [23]. A GLM was applied to evaluate the effects of the pesticides on *L minor* and *D. magna*. The total number of leaves, the total biomass, and the total chlorophyll of *L. minor*, and the percentage of mortality of *D. magna*, were used as the response variables against each concentration (treatment) of the pesticides. In another GLM, the effects of the pesticides and their photodegradation products were evaluated using the same response variables against the type of pesticide (pesticide vs. by-products) and the different concentrations (control vs. the highest concentration of each pesticide and by-product). The GLMs were fitted assuming an adequate error distribution using the *stats* package [23], i.e., Poisson error distribution for number of leaves, Gaussian error distribution for biomass and total chlorophyll, and binomial error distribution for percentage of mortality. Significant differences were evaluated by a pairwise comparison test using the *emmeans* package [24].

For the mortality percentage of *D. magna* only, the lethal concentrations (LC_50_, LC_20_, and LC_10_) for difenoconazole and atrazine were calculated using the *drc* package [25]. The lethal concentrations were calculated with a 95% confidence interval.

**Table 1:** Summary of the characteristics and effects of the studied pesticides as well as their photodegradation products after UV irradiation. *m*/*z* refers to the observed mass/charge number ratio. RT indicates the retention time expressed in minutes.

Name	Structure	Formula	*m*/*z*	RT	Family	Effect
Difenoconazole	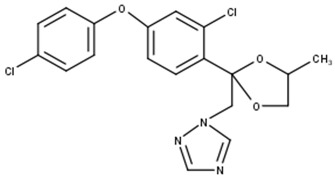	C_19_H_17_Cl_2_N_3_O_3_	405.9	6.9	Triazole	Inhibitor of the biosynthesis of ergosterol in the cellular membrane of fungi [4].
A-Difenoconazole	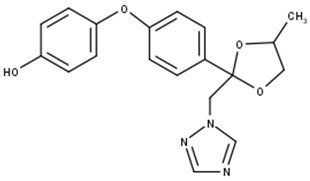	C_19_H_19_N_3_O_4_	354.9	2.9	Triazole	Affects the normal development of aquatic vegetation and reduces chlorophyll production. Affects the mortality of invertebrates (in this study).
B-Difenoconazole	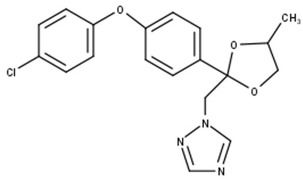	C_19_H_18_ClN_3_O_3_	370.0	5.2	Triazole	Affects the normal development of aquatic vegetation and reduces chlorophyll production. Affects the mortality of invertebrates (in this study).
Atrazine	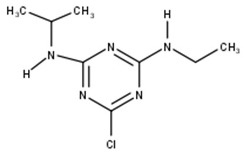	C_8_H_14_ClN_5_	215.7	19.0	Triazine	Inhibitors of photosynthetic electron transport [26].
A-Atrazine	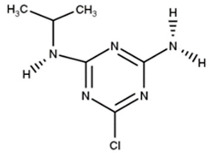	C_6_H_10_ClN_5_	187.0	5.5	Triazine	Affects the normal development of aquatic vegetation and reduces chlorophyll production (in this study).

## 3. Results

### 3.1. Photodegradation of Difenoconazole and Atrazine

After the photodegradation of the pesticides, we identified two photodegradation products for difenoconazole (A-Difenoconazole and B-Difenoconazole) and one for atrazine (A-Atrazine), as shown in Table 1. For A-difenoconazole and B-Difenoconazole, the molecular formulas are C_19_H_19_N_3_O_4_ and C_19_H_18_ClN_3_O_3,_ respectively, while for A-Atrazine, the molecular formula is C_6_H_10_ClN_5_. The three photodegradation products were identified at the following retention times: A-Difenoconazole at 3 min, B-Difenoconazole at 5 min, and A-Atrazine at 5.5 min (Table 1).

### 3.2. Toxicity Tests with L. minor

**Figure 1:**
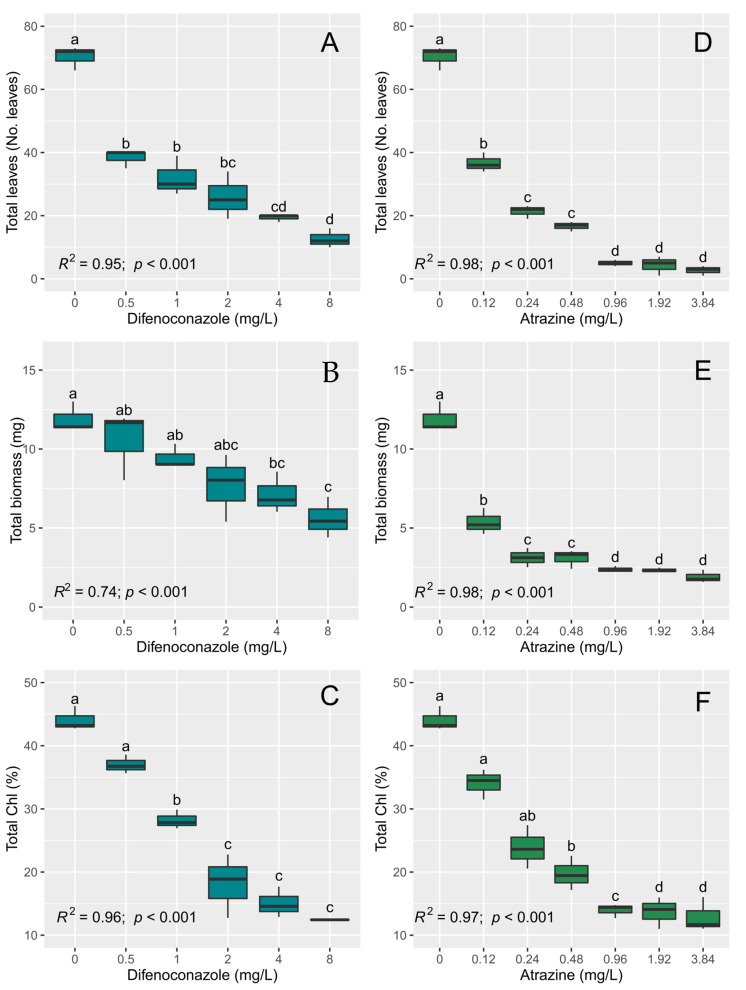
Effects of difenoconazole (**A**–**C**) and atrazine (**D**–**F**) after seven days of exposure on the number of leaves, total biomass, and total chlorophyll (Chl) content of *Lemma minor*. Different lowercase letters denote significant differences in the means at *p* ≤ 0.05 (pairwise comparisons) between different pesticide concentrations.

Both difenoconazole and atrazine have a significant effect on *L. minor* (Table 2). The number of leaves decreases significantly as the concentrations of both pesticides increase, as shown in Figure 1. For difenoconazole, the highest number of leaves is observed in the control, which is different from the other concentrations, while there are no significant differences in the number of leaves between the concentrations of 0.5 and 1 mg/L and between the concentrations of 2, 4, and 8 mg/L (Figure 1). Regarding biomass, the highest biomass is observed in the control, and there are no significant differences between the concentrations of 0.5 and 1 mg/L; however, there are significant differences for the higher concentrations, with the concentration of 8 mg/L of difenoconazole having a greater effect on the biomass of *L. minor* (Figure 1). The total content of chlorophyll is significantly higher in the control and in the concentration of 0.5 mg/L of difenoconazole, and it decreases with higher concentrations, with the concentration of 8 mg/L of difenoconazole being the dose with the greatest effect on the chlorophyll content of *L. minor (*Figure 1).

For atrazine, the highest number of leaves is observed in the control, which is different from the other concentrations, while there are no significant differences in the number of leaves between the concentrations of 0.24 and 0.48 mg/L, and the highest concentrations are the ones that have a negative effect on the number of leaves of *L. minor* (Figure 1). Regarding biomass, the highest biomass is observed in the control. There is a marked significant decrease towards higher concentrations of atrazine, but no significant differences are observed between these concentrations (Figure 1). The total content of chlorophyll is significantly higher in the control and in the concentration of 0.12 mg/L of atrazine, and it decreases towards the highest concentrations, with the concentrations of 0.92, 1.92, and 3.84 mg/L of atrazine being the doses with the greatest effect on the chlorophyll content of *L. minor*, although there are no significant differences between them (Figure 1).

**Figure 2:**
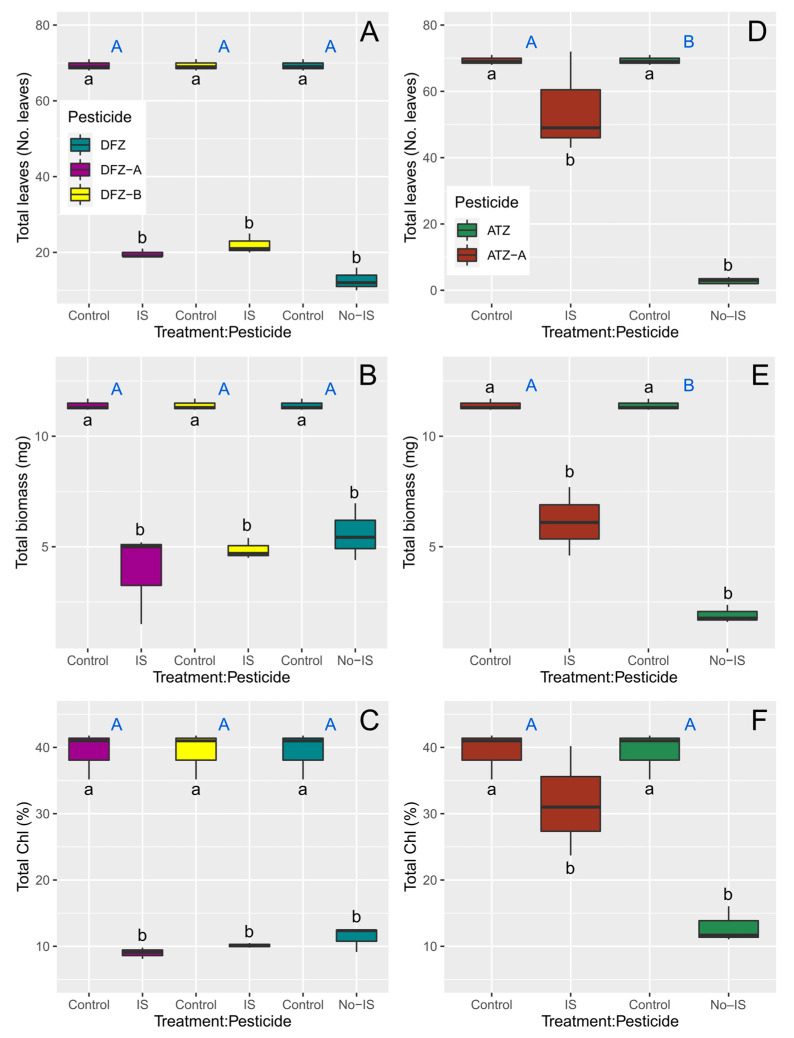
Effects of difenoconazole (**A**–**C**) and atrazine (**D**–**F**) and their photodegradation products after seven days of exposure on the number of leaves, biomass, and total chlorophyll (Chl) content of *L. minor*. Control refers to a concentration of 0 mg/L, whereas IS (irradiated solution) refers to highest concentration used in the bioassays, i.e., 8 mg/L for difenoconazole and its photodegradation products, and 3.84 mg/L for atrazine and its photodegradation product. Different letters denote significant differences in the means at *p* ≤ 0.05 (pairwise comparisons) between different treatment (blue uppercase letters) and pesticide concentrations (lowercase letters).

In general, significant differences are found for the effects of different concentrations (control vs. highest concentration) of both pesticides and their photodegradation products on *L*. *minor*, but some exceptions are observed in the response of *L*. *minor* between the type of pesticide (e.g., Atrazine vs. A-Atrazine) (Table 3). For difenoconazole, in terms of number of leaves, biomass, and total chlorophyll content, both the pesticide and its two photodegradation products have the same effect (Figure 2).

For atrazine, there are significant differences in the effects of the pesticide and its photodegradation product on the number of leaves, biomass, and total chlorophyll content of *L. minor* (Figure 2).

### 3.3. Toxicity Tests with D. magna

**Figure 3:**
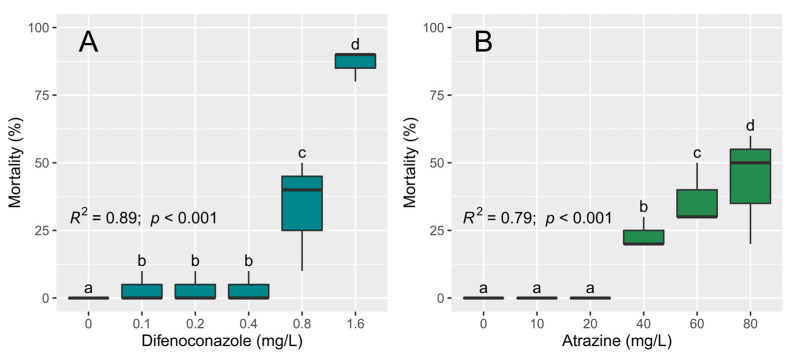
Effects of difenoconazole (**A**) and atrazine (**B**) after 48 h of exposure on the percentage of mortality of *Daphnia magna*. Different lowercase letters denote significant differences in the means at *p* ≤ 0.05 (pairwise comparisons) between different pesticide concentrations.

Both difenoconazole and atrazine have a significant effect on *D. magna* neonates (Table 2). For difenoconazole, in the control, no mortality is observed in the *D. magna* neonates, while neonatal mortality is significantly higher at the concentrations of 0.8 and 1.6 mg/L of difenoconazole (Figure 3). For atrazine, no mortality of *D. magna* neonates is observed in the control nor in the concentrations of 10 and 20 mg/L. At 40, 60, and 80 mg/L concentrations of atrazine, a significant increase in the mortality of *D. magna* neonates is observed (Figure 3).

**Figure 4:**
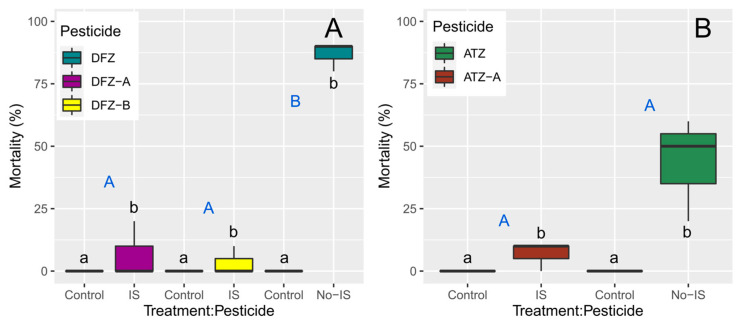
Effects of difenoconazole (**A**) and atrazine (**B**) and their photodegradation products after 48 h of exposure on the percentage of mortality of *D. magna*. Control refers to a concentration of 0 mg/L, whereas IS (irradiated solution) refers to highest concentration used in the bioassays, i.e., 1.6 mg/L for difenoconazole and its photodegradation products, and 80 mg/L for atrazine and its photodegradation product. Different letters denote significant differences in the means at *p* ≤ 0.05 (pairwise comparisons) between different treatment (blue uppercase letters) and pesticide concentrations (lowercase letters).

In general, significant differences are found for the effects of different concentrations (control vs. highest concentration) of both pesticides and their photodegradation products on *D. magna*, but some exceptions are observed in the mortality response of *D. magna* neonates between the type of pesticide (e.g., Difenoconazole vs. A-Difenoconazole) (Table 3). For difenoconazole, there is a significant difference between the pesticide and its photodegradation products, with neonatal mortality being higher with the pesticide than with the two photodegradation products (Figure 4). In the case of atrazine, there is no significant difference in *D. magna* neonate mortality between the pesticide and its photodegradation product (Figure 4).

Regarding lethal concentrations of difenoconazole, the dose that is necessary to cause death in 50% of individuals is LC_50_—48 h = 0.97 mg/L (0.85–1.08, 95% confidence interval). LC_20_—48 h and LC_10_—48 h values are determined for 0.66 mg/L (0.55–0.77) and 0.53 mg/L (0.41–0.65) of difenoconazole, respectively (Figure S1).

For atrazine, the LC_50_—48 h value is 86.19 mg/L (66.04–106.34), being higher than the highest concentration evaluated in this study (80 mg/L). The dose to cause death in 20% and 10% of *D. magna* are LC_20_—48 h = 40.63 mg/L (30.62–50.64) and LC_10_—48 h = 26.17 mg/L (15.28–37.06) of atrazine, respectively (Figure S1).

## 4. Discussion

These results show how the two pesticides, difenoconazole and atrazine, and their photodegradation products cause a toxic effect on the growth of *L. minor* and on the mortality of *D. magna* neonates. We found that pesticides in their original state are a serious threat to aquatic biota and that photodegradation products remain toxic to aquatic organisms even though they are degraded. Our findings show that the number of leaves, biomass, and chlorophyll content of *L. minor*, as well as the mortality of *D. magna*, are sensitive metrics to quantify the toxic effects of pesticides and photodegradation products in aquatic environments. We believe that atrazine is a pesticide with a broad toxicological spectrum for plants and low toxicity for crustaceans at <20 mg/L concentrations in aquatic environments, whereas difenoconazole shows low toxicity for plants and high toxicity for crustaceans from 0.1 mg/l concentrations. Therefore, our results complement the information presented in other studies, where no effects of atrazine on *D. magna* was found [3,27,28]. In the research of Klematová et al. [3], they carried out a homogeneous photocatalytic degradation of atrazine through the photo-Fenton system and toxicity tests on *D. magna* and *L. minor*, showing that atrazine was toxic for *L. minor*, but it did not affect *D. magna*. Consequently, they established that photocatalytic degradation reduces the negative effect of atrazine on *D. magna*, while photodegradation products still negatively affect the growth of *L. minor*. Here, we demonstrated that when using a pesticide of different nature, aquatic biota may also response using different pathways.

In the case of difenoconazole, the work carried out by Man et al. [9] supports our results. They photodegraded difenoconazole in water as well as in soil, and they concluded that the toxicity caused in *D. magna* by the photodegradation products is significantly lower than the parent compound difenoconazole. Other researchers evaluated *D. magna* against difenoconazole and determined that there are effects on antioxidant and detoxifying enzymes, and on the lipid peroxidation of crustacean [4].

The fact that we could establish the toxicity that these pesticides may cause in aquatic biota provides us the context of what happens to these chemicals after they are applied to crops. It is known that the transformation of pesticides under different processes (photodegradation, hydrolysis, photolysis, physical-chemical conditions, degradation by microorganisms, etc.) produces new compounds that are released into the environment; however, we do not fully know the role these compounds play in the ecosystem.

The approach that we describe in this work allows us to evaluate the status of two common species that are part of the aquatic biota and that are known as indicators of ecotoxicity caused by pesticides that are used worldwide. Toxicological assays using standard indicators are useful, but complementary tests using autochthonous organisms would ideally improve the understanding of how pesticides affect local aquatic biota and provide better clues for regulating pesticide applications.

The concentrations of the pesticides and their degraded products assessed in this study may be used as reference information to establish potential hazardous effects in natural water bodies, as stated by Lamkhanter et al. [29]. To complement this study, we suggest performing assays to evaluate the combined effects of pesticides and their photodegradation products e.g., [3], or the combined effects of pesticides with other pollutants, such as microplastics. Additionally, the application of other bioindicators, such as fish, macroinvertebrates, cyanobacteria, or algae, is recommended in order to better understand how pesticides may affect natural ecosystems [30,31]. Another future approach can be the implementation of longer exposure times to toxicants (chronic toxicity) since pesticides can actually be present in the environment for very long periods, and organisms can experience chronic effects from exposure to pesticides and their degraded products [31].

Currently, pesticides and their degradation products have been identified in air, water, and soil in all geographic regions, including those that are very remote from the original site of their environmental release [32]. Knowing the agricultural practices in some countries, such as Ecuador, where banana and sugar cane plantations (crops commonly using difenoconazole and atrazine) were the crops with the largest irrigated area compared to the planted area in 2020, with 91.5% and 94.4%, respectively [17], displays a snapshot of current agricultural practices, where pesticides and their degradation products are potentially transported by irrigation systems to aquatic ecosystems, thereby contaminating water resources. For instance, Ochoa-Cueva et al. [20] reported that several sites in southern Ecuador present a high risk of pesticide exposure due to the indiscriminate application of pesticides across croplands. We recommend that in areas seriously exposed to pesticides, an irrigation water treatment based on photodegradation or advanced oxidation processes [33] may minimize the negative impacts of pesticides to the environment. However, as we found that degraded pesticide products can remain toxic in water, it is highly recommended the application of organic farming and the restriction of the use of pesticides, or only allowing those that degrade quickly and are less harmful to the ecosystem [34].

## 5. Conclusions

The results of the present investigation confirm that number of leaves, biomass, and chlorophyll content of *L. minor* and the mortality of *D. magna* are sensitive response variables that can be used to determine the toxicity of pesticides and photodegradation products in aquatic environments.

Pesticides, such as difenoconazole and atrazine, are a serious threat to aquatic biota and, after they are degraded by, for example, UV exposure, the resulting compounds remain toxic in the environment.

## Figures and Tables

**Table 2:** Summary of the GLM for the number of leaves, biomass, and total chlorophyll of *L. minor* and for the percentage of mortality of *D. magna* after being exposed to different concentrations (treatments) of difenoconazole and atrazine pesticides. Significant differences are indicated with the *p*-values in bold.

Species	Pesticide	Variable	Source of Variation	Df	*F*	*p*
*Lemna minor*	Difenoconazole	No. of leaves	Treatments	12	34.38	**<0.001**
		Biomass	Treatments	12	6.95	**0.003**
		Total chlorophyll	Treatments	12	70.64	**<0.001**
						
	Atrazine	No. of leaves	Treatments	12	67.94	**<0.001**
		Biomass	Treatments	12	103.83	**<0.001**
		Total chlorophyll	Treatments	12	72.20	**<0.001**
						
*Daphnia magna*	Difenoconazole	Mortality	Treatments	12	37.09	**0.001**
						
	Atrazine	Mortality	Treatments	12	11.92	**<0.001**

**Table 3:** Summary of the GLM for the number of leaves, biomass, and total chlorophyll of *L. minor* and for the percentage of mortality of *D. magna* after being exposed to different concentrations (treatments) of pesticides and their photodegradation products (pesticides). Significant differences are indicated with the *p*-values in bold.

Species	Pesticide	Variable	Source of Variation	Df	*F*	*p*
*L. minor*	Difenoconazole	No. of leaves	Treatments	16	288.10	**<0.001**
			Pesticides	14	0.82	0.443
		Biomass	Treatments	16	25.02	**<0.001**
			Pesticides	14	0.13	0.874
		Total chlorophyll	Treatments	16	165.13	**<0.001**
			Pesticides	14	0.08	0.920
						
	Atrazine	No. of leaves	Treatments	10	104.38	**<0.001**
			Pesticides	9	41.89	**<0.001**
		Biomass	Treatments	10	78.27	**<0.001**
			Pesticides	9	6.40	**0.032**
		Total chlorophyll	Treatments	10	16.80	**0.003**
			Pesticides	9	5.07	0.051
						
*D. magna*	Difenoconazole	Mortality	Treatments	10	9.24	**0.009**
			Pesticides	9	6.60	**0.010**
						
	Atrazine	Mortality	Treatments	10	8.69	**0.016**
			Pesticides	9	4.67	0.060

## Data Availability

The data presented in this study are available from the corresponding author upon request. The data are not publicly available due to further analyses and assays with the data used here.

## Supplementary Materials

The following supporting information can be downloaded at https://www.mdpi.com/article/10.3390/toxics11030213/s1, Table S1: Formulation for *Lemna minor* nutrient solution according to the Standard Methods 8211 [11]. A, B, and C refer to the stock solution to be prepared. Table S2: Formulation for preparing reconstituted freshwater for *Daphnia magna* according to the Standard Methods 8010:I [11]. Figure S1. Dose–response relationships between the percentage of mortality of *Daphnia magna* neonates and the concentrations of difenoconazole (**A**) and atrazine (**B**).
