# Supplementary material for: Toxicity of Difenoconazole and Atrazine and Their Photodegradation Products on Aquatic Biota: Environmental Implications in Countries Lacking Good Agricultural Practices
Source: Toxics. 2023 Feb 24;11(3):213. doi: 10.3390/toxics11030213 (PMC10051296; doi:10.3390/toxics11030213)
Supplement: Supplementary file 1 [file toxics-11-00213-s001.zip › toxics-2184172-supplementary.pdf]

Supplementary Materials: Toxicity of Difenoconazole and Atrazine and Their Photodegradation Products on Aquatic Biota: Environmental Implications in Countries Lacking Good Agricultural Practices

Julia Mendieta Herrera, Carlos Iñiguez Armijos, Daniel Rosado Alcarria and Silvio Aguilar Ramírez

Table S1. Formulation for Lemna minor nutrient solution according to Standard Methods 8211 [11]. A, B, and C refers to the stock solution to be prepared.

Solution	Stock Solution Concentration
**A:**	
$NaNO_3$	25.5 g/L
$NaHCO_3$	15.0 g/L
K_2HPO_4	1.04 g/L
**B:**	
$CaCl_2 \cdot 2H_2O$	4.41 g/L
$MgCl_2$	5.7 g/L
$FeCl_3$	0.096 g/L
$Na_2EDTA \cdot 2H_2O$	0.3 g/L
$MnCl_2$	0.264 g/L
**C:**	
$MgSO_4 \cdot 7H_2O$	14.7 g/L
H_3BO_3	0.186 g/L
$Na_2MoO_4 \cdot 2H_2O$	7.26 mg/L
$ZnCl_2$	3.27 mg/L
$CoCl_2$	0.78 mg/L
$CuCl_2$	0.009 mg/L

To prepare this nutrient solution, add 1mL of each stock solution to 100 mL deionized water. Adjust to pH 7.5 – 8.0.

Table S2. Formulation for preparing reconstituted freshwater for Daphnia magna according to Standard Methods 8010:I [11].

Water Type	Salts Required in mg/L				Water quality		
	$NaHCO_3$	$CaSO_4$. $2H_2O$	$MgSO_4$	KCl	pH	Hardness mg $CaCo_3$/L	Alkalinity mg $CaCO_3$/L
Very soft	12	7.5	7.5	0.5	6.4 - 6.8	10-13	10-13
Soft	48	30	30	2.0	7.2 - 7.6	40-48	30-35
Moderately hard	96	60	60	4.0	7.4 - 7.8	80-100	60-70
Hard	192	120	120	8.0	7.6 - 8.0	160-180	110-120
Very hard	384	240	240	16.0	8.0 - 8.4	280-320	225-245

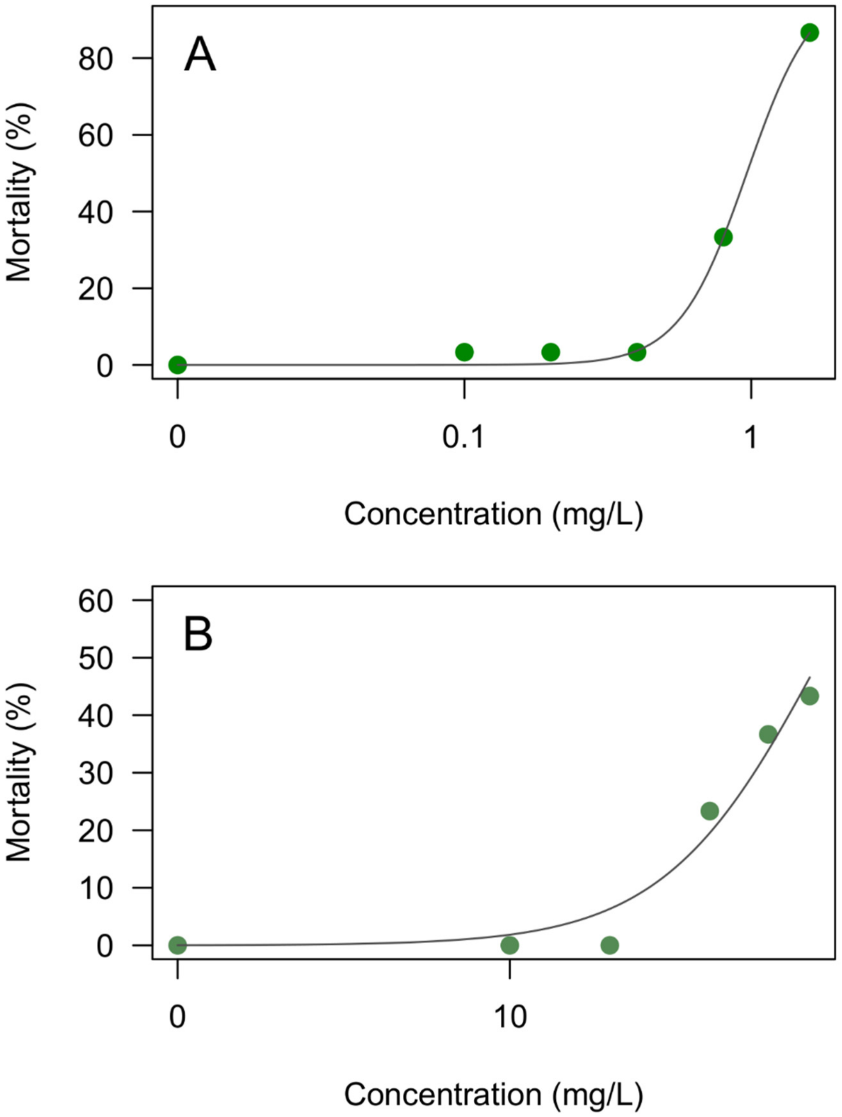

Figure S1. Dose-response relationships between the percentage of mortality of Daphnia magna neonates and the concentration of difenoconazole (A) and atrazine (B).
